# Enhancing the Discrimination Ability of a Gas Sensor Array Based on a Novel Feature Selection and Fusion Framework

**DOI:** 10.3390/s18061909

**Published:** 2018-06-12

**Authors:** Changjian Deng, Kun Lv, Debo Shi, Bo Yang, Song Yu, Zhiyi He, Jia Yan

**Affiliations:** 1 College of Electronic and Information Engineering, Southwest University, Chongqing 400715, China; deng150911@email.swu.edu.cn (C.D.); ny4501yb@email.swu.edu.cn (B.Y.); s0531@email.swu.edu.cn (S.Y.); hzy563255@email.swu.edu.cn (Z.H.); 2 High Tech Department, China International Engineering Consulting Corporation, Beijing 100048, China; lvk@ciecc.com.cn; 3 Westa College, Southwest University, Chongqing 400715, China; shidebo@email.swu.edu.cn

**Keywords:** electronic nose, feature selection, feature fusion, multiclass recognition, sensor drift

## Abstract

In this paper, a novel feature selection and fusion framework is proposed to enhance the discrimination ability of gas sensor arrays for odor identification. Firstly, we put forward an efficient feature selection method based on the separability and the dissimilarity to determine the feature selection order for each type of feature when increasing the dimension of selected feature subsets. Secondly, the K-nearest neighbor (KNN) classifier is applied to determine the dimensions of the optimal feature subsets for different types of features. Finally, in the process of establishing features fusion, we come up with a classification dominance feature fusion strategy which conducts an effective basic feature. Experimental results on two datasets show that the recognition rates of Database I and Database II achieve 97.5% and 80.11%, respectively, when *k* = 1 for KNN classifier and the distance metric is correlation distance (COR), which demonstrates the superiority of the proposed feature selection and fusion framework in representing signal features. The novel feature selection method proposed in this paper can effectively select feature subsets that are conducive to the classification, while the feature fusion framework can fuse various features which describe the different characteristics of sensor signals, for enhancing the discrimination ability of gas sensors and, to a certain extent, suppressing drift effect.

## 1. Introduction

An artificial olfactory system (AOS), also known as the machine olfactory system or electronic nose (E-nose), is designed for imitating the biological sensory system based on the principle of bionics. Nowadays, it has become a major innovation in the field of gas detection technology, due to its advantages, such as real time, non-invasiveness, easy operation, and low cost. However, there are still some deficiencies in AOS, such as being susceptible to the environment, not directly distinguishing the mixed gas, and drifting over time. Since the concept of E-nose was put forward in 1994 [1], the perception and judgment process of bionic olfactory information, as well as its related applications, have been of wide concern to scholars in related fields [2,3,4,5,6].

On the one hand, it is observed that some features of chemical sensors may not be necessary, and only a subset of the original features contribute to the classification when deploying a gas sensor array for a specific application [7]. Meanwhile, the cross-sensitivity of sensor array has both merits and demerits. Specifically, this cross-sensitivity is conducive to the detection of various gases when the number of sensors is limited. However, it also leads to redundancies and interferences. The sensor array may produce redundant, incomplete, imprecise, and inconsistent information, and the presence of these irrelevant features increases the dimensionality of the feature space, which may reduce the accuracy of the pattern recognition. Robust features can describe the characteristic of sensor signals effectively. The performances of classifiers can be improved by using a subset of features instead of the whole set. This requires a systematic or structured approach to select the optimal subset of sensors to enhance the performance of the overall system.

Generally, feature selection algorithms can be roughly divided into two major categories [8]. The filter approach filters the redundant features by a certain figure of merit using a preprocessing process, such as using the Mahalanobis distance between response distributions to evaluate the configurations of sensor array [9]. It possesses less amount of computation, and can find feature sets which are able to be applied to models with different requirements. The wrapper approach selects feature subsets by models trained on all the feature subsets to predict the accuracy of the features. It is more accurate to a certain calibration model [10]. The utilization of classifiers (such as SVM) to select the optimal features of the different sensors for enhancing the classification ability of the compound is a typical wrapper approach [11]. The two approaches are widely used in the feature selection of gas sensor arrays [12,13,14,15]. However, there are still disadvantages of the existing approaches. The filter approach is not accurate enough for different classification models, while the wrapper approach requires more computational resources, and may not have such accuracy in other occasions for its specificity.

Many previous studies have made improvements based on the two basic methods in different research fields. The minimal-redundancy maximal-relevance (mRMR) method is based on the filter method, which selects relevant and nonredundant features according to the mutual information criterion [16]. Sequential forward selection (SFS) has been used for evaluation of breath alcohol measurement [17]. It starts with an empty subset and sequentially adds the best features, which can make the rank of the feature subset higher. Conversely, sequential backward selection (SBS) reduces feature elements sequentially from all to none, and has been used to assess the odor of automobile interior components [18]. In addition, other improved feature selection methods have been also widely applied in many areas, including cloud computing [19], identifying different kinds of meat [20], and social image annotation [21], but still there were not enough such improvements in the E-nose area. However, the improvements mentioned above cannot both reduce the dimension of features and select features efficiently.

On the other hand, it is well known that a single feature cannot fully reflect the characteristics of sensor signals, which achieves low classification accuracy. Thus, the fusion strategy [22] is an advisable choice to improve the prediction accuracy of a gas sensor array. The fusion strategy is an idea that synthesizes the signals from different sources to obtain a better model representation. The purpose of data fusion is to combine information obtained from multiple sources by different strategies, which can potentially achieve a better description and enhance the classification accuracy [23]. Many studies have reported that combining the features of the E-nose, E-tongue, or E-eye will improve the performance of a gas sensor array for odor identification. Hong et al. [24] described the use of four fusion approaches for an E-nose and an E-tongue to distinguish cherry tomato juice from adulteration, and demonstrated that the utilization of perceptual knowledge from both the E-sensors could perform better than using E-nose or E-tongue individually. Buratti et al. [25] proposed an effective mid-level data fusion method to discuss the applicability of the E-nose, E-eye, and E-tongue for the quality decay assessment and characterization of olive oil, which evidenced the ability to classify samples, and has greatly improved the KNN classification model. Rodriguez-Mendez et al. [26] proposed a method of combining the correlations between the chemical parameters from an E-nose and an E-tongue associated with the oxygen and the polyphenolic composition of red wines, which significantly improved the quality of the predictions. In Ref. [23], a multilevel fusion strategy framework of the E-nose and E-tongue is proposed in this paper, which can improve the tea quality prediction accuracies through modeling decision fusion and feature fusion. However, the aforementioned conventional feature fusion strategy focuses on simply and directly combining the original features from different instruments into one feature matrix, which only increases the dimensions of the feature matrix without taking the contribution of each kind of feature on the final classification into account. Recently, Lijun Dang et al. [27] proposed a weighted fusion framework with logarithmic form, which concentrated on the contribution of each feature. However, for the particularity of logarithms, the method of calculating the weights cannot properly reflect the classification accuracy of each feature. 

In this paper, we present a feature selection and fusion framework, and the merits of this paper include the following.

(1) We propose a feature selection method, which couples the filter and wrapper strategies, to evaluate the subfeatures of a gas sensor array using two indicators, i.e., separability and dissimilarity, as well as the KNN classifier, for effectively describing the characteristics of different odors under the premise of reducing the data redundancy as much as possible.
(2) We propose a weighted feature fusion framework combining information according to a classification dominance strategy, for achieving better description of odor and increasing the accuracy of final classification.
(3) The novel feature selection and feature fusion framework can not only improve the recognition rate of a gas sensor array, but also greatly suppress the negative effects of sensor drift effect on gas identification.

In the rest of this paper, we will firstly introduce the whole methodology of the proposed feature selection and fusion framework will be described in Section 2; then, the data sets are introduced briefly in Section 3; the results of this experiment will be shown in Section 4; finally, we will draw our conclusions in Section 5.

## 2. Methodology

**Figure 1:**
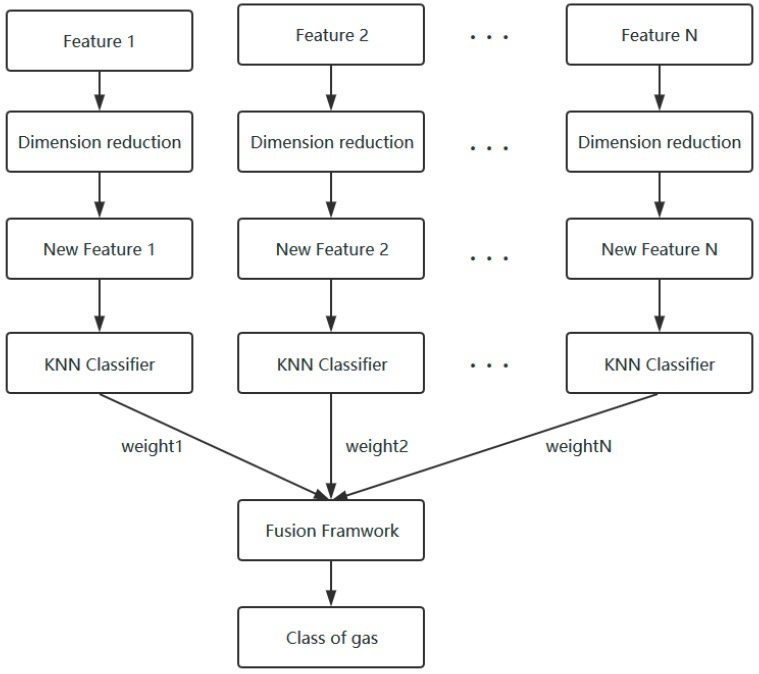
The flow chart of the feature selection and fusion framework.

In this section, a novel feature selection and fusion framework of E-nose are described, which contains three parts: firstly, the separability and dissimilarity of different features are calculated for the order of feature selection. Secondly, use the classifier to determine the optimal dimension of the feature subsets. Finally, fuse the selected features based on a weighted voting according to a classification dominance strategy for obtaining gas classification results. The flow chart of this feature selection and fusion framework is shown in Figure 1.

### 2.1. Feature Selection

In order that the information provided by an E-nose can represent the characteristics of different odors more clearly and can contribute to the classification, a new feature selection method is defined based on the separability and the dissimilarity. First, we introduce the principle of class separability criterion in Section 2.1.1. Then, to eliminate redundant features, we define a dissimilarity criterion in Section 2.1.2. At last, the feature selection algorithm is shown in Section 2.1.3.

#### 2.1.1. Separability Index

A pretty good classification rate will be achieved if one feature produces a distinct scent fingerprint in the feature space for different gases. That is to say, if scent fingerprints contain good separable information, the pattern recognition algorithm can easily identify them. On the contrary, the classification performance will not improve in case all the features have poor information that four classes of odors cannot be correctly identified.

Suppose K is the number of samples for each class of gases, M is the number of dimensions of original feature matrix, and N is the number of classes of gases. $X_{mn}(i)$ denotes the feature of the i-th (i=1,2,⋯,k) sample of the m-th (m=1,2,⋯,M) dimension of the feature matrix (denoted as $f_{m}$) for the n-th (n=1,2,⋯,N) gas (denoted as $G_{n}$). Thus, the mean vector $\mu_{mn}$ for feature $f_{m}$ and gas $G_{n}$ is

(1) $$\mu_{mn}=\frac{\sum_{i=1}^{K}X_{mn}(i)}{K}.$$

The Euclidean distance between each sample of each dimension of feature matrix for each class of gases and the mean vector can be written as

(2) $$d_{mn}(i)=\left\|X_{mn}(i)-\mu_{mn}\right\|.$$

The mean and variance of $d_{mn}(i)$ for feature $f_{m}$ and gas $G_{n}$ are defined as Equations (3) and (4):

(3) $$\mu_{d_{mn}}=\frac{\sum_{i=1}^{K}d_{mn}(i)}{K},$$

(4) $$\sigma_{d_{mn}}^{2}=\frac{\sum_{i=1}^{K}{\left(d_{mn}(i)-\mu_{d_{mn}}\right)}^{2}}{K-1}.$$

Then $\sigma_{m1}^{2}$, defined as the average of $\sigma_{d_{mn}}^{2}$ for each gas as Equation (5), is a measure of variation of within-class scatter for feature $f_{m}$:

(5) $$\sigma_{m1}^{2}=\frac{\sum_{n=1}^{n}\sigma_{d_{mn}}^{2}}{N}.$$

Define the sample mean vector for *N* classes of gases as follows:

(6) $$\mu_{m}=\frac{\sum_{n=1}^{N}\mu_{mn}}{N}.$$

The Euclidean distance from $\mu_{mn}$ to overall mean vector $\mu_{m}$ is:

(7) $$d_{mn}=\left\|\mu_{mn}-\mu_{m}\right\|.$$

The mean and variance of $d_{mn}$ are

(8) $$\mu_{d_{m}}=\frac{\sum_{n=1}^{N}d_{mn}}{N},$$

(9) $$\sigma_{m2}^{2}=\frac{\sum_{n=1}^{N}{\left(d_{mn}-\mu_{d_{m}}\right)}^{2}}{N-1}.$$

Here, $\sigma_{m2}^{2}$ is a measure of variation of between-class scatter for feature $f_{m}$.

Finally, we define the ratio of $\sigma_{m2}^{2}/\sigma_{m1}^{2}$ as the class separability index (*SI*):

(10) $$SI(f_{m})=\sigma_{m2}^{2}/\sigma_{m1}^{2}.$$

Hence, $SI(f_{m})$ describes the capability of class separability for the features to be selected. The larger $SI(f_{m})$ means the more separability information that the feature contains.

#### 2.1.2. Dissimilarity Index

We can acquire little additional information if different features are similar for all the gases. It means that each of the selected features not only must have good separability, but also contain more diverse but less redundant information for a subset of features to be optimized, so we define a dissimilarity index (DI) for all the features as follows:

(11) $$DI(f_{i},f_{j})=1-\left|\rho (f_{i},f_{j})\right|\;(i,j=1,2,\cdots ,M),$$

where $\rho (f_{i},f_{j})$ is the correlation coefficient between the feature $f_{i}$ and $f_{j}$. The larger $DI(f_{i},f_{j})$ means there is less shared information between two features. It means that the selected features have more additional, but less redundant information for classification. 

#### 2.1.3. Feature Selection Algorithm

The purpose of class separability and dissimilarity is to choose the optimal feature subsets for classification. The larger both separability and dissimilarity means the selected features have more advantages to enhance classification capability. The steps are described in detail in the following Algorithm 1.
**Algorithm 1.** Feature Selection**Input:**  Original feature matrix $X_{M}$ with *M*-dimensional features. **Output:**  Selected feature subset S with *D*-dimensional features (D=1,2,⋯,M). **Procedure:**  1: D = 1. Compute $SI(f_{i})\;(i=1,2,\cdots ,\;M)$ of each dimension of the original feature matrix and record the *score*_1_: $score_{1}=SI(f_{i})$. Choose the feature with the largest $score_{1}$ as the first element of the optimal feature subset S. Then, the remaining feature element is $X_{M-D}$.  2: **do**  Step 1: D = D + 1. Then, choose a feature element from $X_{M-D}$ in turn, and combine the element with S into a new feature subset $X_{T}$, all subset $X_{T}$ make up a new feature matrix $X^{\prime }$. Compute the class separability index (SI) of each feature subset in the $X^{\prime }$ and the SI is defined as $SI=\frac{2}{D}\sum_{i=1}^{D}SI(f_{i})$.  Step 2: For the formed new feature matrix $X^{\prime }$ in Step 1, obtain $t=\left(\begin{array}{l} 2\\ D \end{array}\right)$ subsets. Then, compute the average DI of the pairwise dissimilarity of all the subsets.  Step 3: For each subset, compute the *score*_2_ defined as $score_{2}=SI+DI$, which reflects whether the feature subset is appropriate.  Step 4: Put the feature element with the largest value of $score_{2}$ into S and reset the remaining feature element $X_{M-D}$.  Step 5: Input the selected feature subset S with *D*-dimensional features into the classifier. Then, the classification accuracy of the *D*-dimensional features accuracy(D) will be obtained.  **End while** until the number of selected elements *D* reaches M.  3: Choose the best classification accuracy from accuracy(D)(D=1,2,3 ⋯ M) as the final accuracy for this kind of feature after feature selection. If accuracy(i)=accuracy(j) but i≤j(i,j=1,2,3 ⋯ M), i can be considered as the optimal feature dimension. **Return:**
S = {s^1^, s^2^, …, s^M^}. **Note:** The larger *score*_2_ means the feature is more beneficial to increasing classification performance.


### 2.2. Feature Fusion Framwork

Suppose that there are L kinds of features and N types of samples. Each kind of feature makes decisions according to its prediction accuracy on test data. Firstly, each kind of feature is used as the input of the classifier, respectively, and L classification accuracy rates are obtained and denoted as $a=[a_{1},a_{2},\cdots ,a_{L}]$. The importance weight of each kind of feature $w=[w_{1},w_{2},\cdots w_{L}]$ is calculated by Equation (15):

(12) $$w_{i}=\frac{a_{i}}{\sum_{i=1}^{L}a_{i}},$$

where $w_{i}(i=1,2\cdots ,L)$ denotes the importance weight of the *i*-th kind of feature.

For each sample, the output form of the classifier for the *L* kinds of features can be predicted as

(13) $$\delta ={\left[\delta_{1},\delta_{2},\cdots ,\delta_{i},\cdots ,\delta_{L}\right]}^{T},$$

where $\delta_{i}\in [1,2,\cdots ,N]$ can be transformed into binary encoding. If the prediction result of the *i*-th kind of feature is $\delta_{i}=1$, then encode it by $\delta_{i}^{binary}={\left[\underset{N\;elements}{\underset{︸}{1\;0\;\cdots \;0}}\right]}^{T}$. Similarly, if $\delta_{i}=2$, then encode it by $\delta_{i}^{binary}={\left[\underset{Nelements}{\underset{︸}{0\;1\;\cdots \;0}}\right]}^{T}$. By that analogy, if $\delta_{i}=c$, its binary encoding $\delta_{i}^{binary}$ is a vector with *N* elements, whose *c*-th element equals 1 and the others are 0. Thus, we can obtain

(14) $$\delta^{binary}={\left[\delta_{1}^{binary},\delta_{2}^{binary},\cdots ,\delta_{i}^{binary},\cdots ,\delta_{L}^{binary\;}\right]}^{T}.$$

The weighted feature fusion framework according to a classification dominance fusion strategy leverages the classification rates of the base features, and makes a final decision based on Equations (15) and (16).

(15) $$fusion=[fusion_{1},fusion_{2},\cdots fusion_{j}\cdots fusion_{N}]=w\cdot \delta^{binary},$$

where $fusion_{j}(j=1,2,\cdots ,N)$ is the fusion score of the *j*-th class. It means that each class has its own fusion score, and the class label of one sample can be predicted by the maximum fusion score, which is shown as

(16) $$predict\_label=\max(fusion_{1},fusion_{2},\cdots fusion_{j}\cdots fusion_{N}).$$

All the computations involved in this paper are implemented in the E-nose software system and Matlab R2015b (Mathworks, Natick, MA, USA). The K-Nearest Neighbor (KNN) algorithm is used as the classifier, which classifies samples based on closest training examples in the feature space. There are numerous advantages of the KNN that has been proved. One of the advantages is that it is effective to reduce the misclassification when the number of samples in the training dataset is large. Meanwhile, KNN can easily deal with multiclass recognition problems especially when the class size is three and higher. What is more important is that KNN is superior to many other supervised learning methods, such as support vector machine (SVM), neural network (NN), etc. Since the process of their parameters to be optimized will cost much time, while the KNN method demands only few parameters to tune for achieving excellent classification accuracy: the value of *k* and the distance metric [28,29,30].

## 3. Description of Experimental Data

In this paper, two different datasets of gas sensor arrays are utilized, and here is a brief description of the materials and gas sensor array to make the paper self-contained.

### 3.1. Dataset I

A sensor array containing fourteen metal oxide sensors (TGS800, TGS813, TGS816, TGS822, TGS825, TGS826, TGS2600, TGS2602, TGS2620, WSP2111, MQ135, MQ138, QS-01, and SP3S-AQ2), and one electrochemical general air quality sensor (AQ) produced by Dart Sensors Ltd. (Exeter, UK) were applied to detect four types of rates wounds (uninfected and infected by *Staphylococcus aureus*, *Pseudomonas aeruginosa*, and *Escherichia coli*, respectively). The details of the samples and experiments are presented in previous publication [31]. The specific distribution of data is shown in Table 1.

Seven kinds of features were extracted from the original response curves and their transform domains: maximum value (MV), the DC component and first order harmonic component of the coefficients of fast Fourier transformation (FFT), and the approximation coefficients of discrete wavelet transformation (DWT) based on wavelets Db1, Db2, Db3, Db4, and Db5, respectively. The structures of the feature matrix are shown in Table 2.

### 3.2. Dataset II

We used a big sensor data array with long-term drift effect of 36 months, which was publicly released by UCI Machine Learning Repository [32], as the second dataset. This data contains 13910 measurements from an E-nose system with 16 gas sensors (TGS2600, TGS2602, TGS2610, and TGS2620 (four of each) from Figaro Engineering Inc. (Tianjin, China). All sensors are exploited to detect six kinds of pure gaseous substances at distinct concentration levels, including ethanol, ethylene, ammonia, acetaldehyde, acetone, and toluene. The concentration ranges of the six kinds of gases are shown in Table 3. Eight kinds of features extracted from the original response data for each sensor make up a 128-dimensional feature vector (16 sensors × 8 features). In total, 10 batches of sensor features are collected at different time intervals. The details of the sensor batches are presented in Table 4.

During the gas injection phase, the resistance of the sensor will increase with the growth trend gradually slowing down, and the response will gradually decrease with the declining trend gradually slowing down during the cleaning phase. Therefore, we can use the maximum/minimum value of the exponential moving average (EMA) to reflect the growth/declining trend of the sensor signals [32], and the EMA is defined as:

(17) y[k]=(1−α)y[k−1]+α(R[k]−R[k−1]),

where α is a scalar smoothing parameter between 0 and 1, while y[k] and R[k] are the EMA and the response at time k, respectively.

Three different values of α(α=0.1,α=0.01,a=0.001) were used in the formula to obtain three different maximum/minimum values of EMAs for increasing stage and decreasing stage, which are defined as EMAi1, EMAi2, EMAi3, EMAd1, EMAd2 and EMAd3, respectively. In addition, another two features contain a steady-state feature, defined as the difference of the maximal resistance change and the baseline (DR); normalized version steady-state feature (NDR), is expressed by the ratio of the maximal resistance and the baseline values. In order to display that the feature selection and fusion framework can enhance the discrimination ability of the gas sensor array with drift effect, batches 1 to 9 are used as training set, while the batch 10 is used as the test set, respectively.

## 4. Results and Discussion

In this work, the different values of *k* which will be tested are {1, 3, 5, 7, 9}, and the different distance metrics are Euclidean distance (EU), cityblock distance (CB), cosine distance (COS) and correlation distance (COR) [28,33].

### 4.1. The Optimal Value of k and the Distance Metrics

First of all, in order to further certify the optimal value of *k* and the distance metrics for different features of two datasets, we perform a comparison between different values of *k* and distance metrics in KNN classifier without feature selection, and each kind of feature is experimented individually. Table 5 and Table 6 lists the classification accuracy of different values of *k* and distance metrics using the KNN classifier for Dataset I and Dataset II, respectively. In Table 5, it is observed that the classification rate descends as the values of *k* increases on the whole. Compared with the feature of MV, the recognition results of the features from transform domain are greatly improved. As for the distance metric, it is obvious that the COS and COR perform generally better than EU and CB. For the COS and COR, the performances of DB1, DB2, DB3, and DB4 can achieve above 90%. For the COR, especially, the best performances obtained by Db1 are better than COS, which can achieve 93.75% when *k* = 1, while the COS has the performance of 90.00%. For Dataset II, which is shown in Table 6, the variation of classification accuracy using the classifier with different values of *k* and distance metrics is inconspicuous, and the performances of different features are all bad. It indicates that the sensor drift effect seriously affects the stability of the outputs of sensors, and finally deteriorates the performance of the classifier. In general, EU and CB measure the absolute distance between points in the high dimensional space, which is directly related to the position coordinates of each point, i.e., the values of the elements of each feature. EU and CB can reflect the absolute difference of individual numerical values of features, so they are usually used to analyze the difference from the numerical values of different dimensions. The COS and COR measure the angle between the vectors and lay emphasis on the difference in the directions of the vectors rather than the positions. For the classification for the signals of a gas sensor array, the patterns of distinct odors are mainly reflected in the relative directions among different features rather than the absolute values. Therefore, COS and COR are superior to EU and CB. To exhibit the effect of feature selection and fusion framework, COS and COR, as well as *k* = 1, are used in the following experiments.

### 4.2. Separability Index and Dissimilarity Matrix

**Figure 2:**
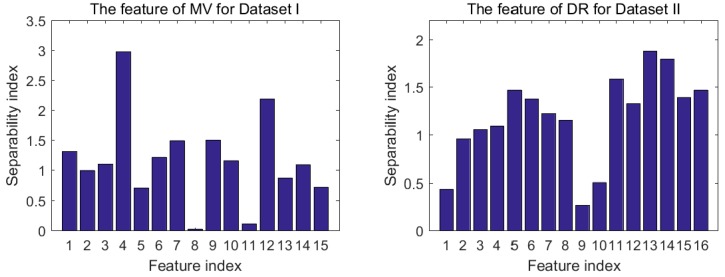
Separability index of maximum value (MV) for Dataset I and difference of the maximal resistance change and the baseline (DR) for Dataset II.

Among all the features of an E-nose, not all of them are sensitive to the target gases. In order to remove the features which are not helpful for classification, the proposed feature selection method is conducted in this section. MV of Dataset I and DR of Dataset II are taken as examples to illustrate the process of feature selection. Figure 2 shows the class separability index of 15 features of MV and class separability index of 16 features of DR. It is obvious that sensor 4 for MV and sensor 13 for DR have the highest separability, while sensor 8 of MV and sensor 9 of DR have the worst one. Hence, if the selection is just based on separability, the feature of sensor 4 for MV and feature of sensor 13 for DR should be chosen in the final subset. 

**Figure 3:**
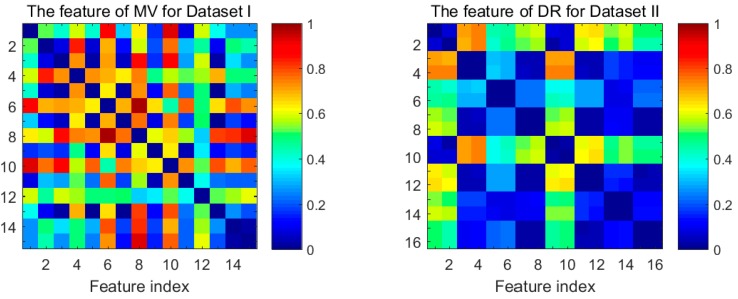
Visualization of the dissimilarity matrix of MV and DR.

The dissimilarity for all the pairwise combinations of features of MV for Dataset I and DR for Dataset II, as computed by Equation (11), are shown in Figure 3. Colors biased towards red/blue tones denote higher/lower values of the dissimilarity. Note that the distributions of values are symmetric with respect to the swap of features for Figure 3. We can see clearly that the combination of features index of the MV with highest dissimilarity turns out (6, 8), and the different situation appears for the DR features when the features index for the selected pair of features is (4, 10). Hence, if we choose two subfeatures only based on dissimilarity, the feature subset {6, 8} of the MV and the feature subset {4, 10} will be chosen on the basis of the pigment band.

### 4.3. Optimal Numbers of Different Kinds of Features after Selection

**Figure 4:**
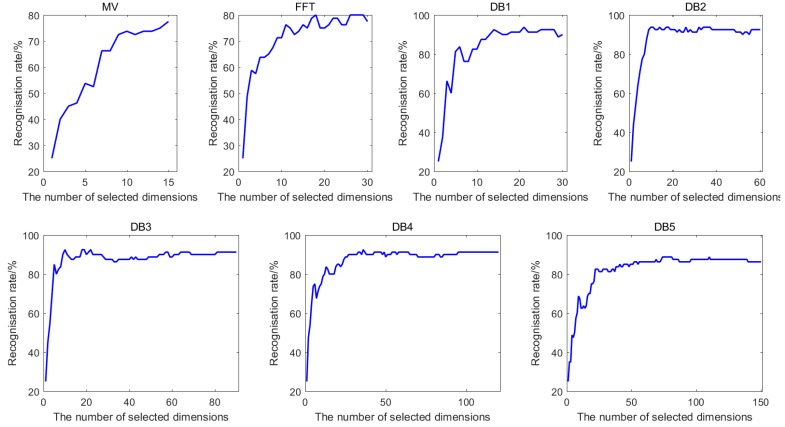
The classification accuracies of seven features for Dataset I.

**Figure 5:**
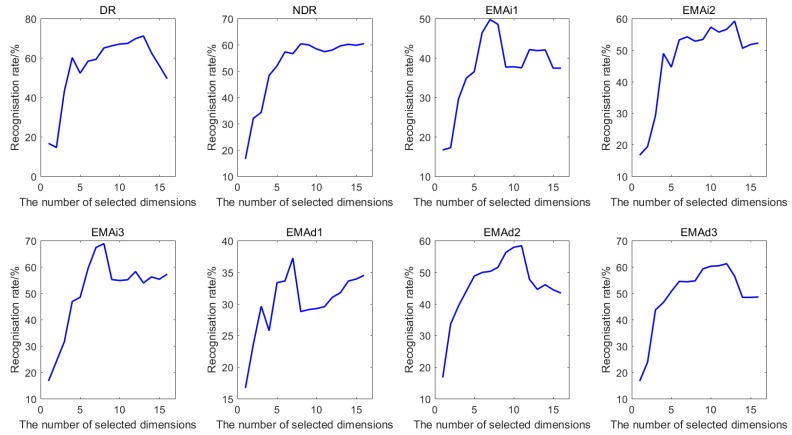
The classification accuracies of eight features for Dataset II.

To obtain the optimal numbers of different features after feature selection, the classification accuracies performed by KNN classifier with the selected feature subset for Dataset I and Dataset II are computed. Figure 4 shows the best classification accuracies of seven features of Dataset I, while Figure 5 shows the best classification accuracies of eight features of Dataset II when COS is used as distance metric and *k* = 1. It can be clearly observed that the recognition rate rises rapidly when the number of selected feature dimensions, which are put into KNN classifier, is small. Generally, the recognition rate increases with the increasing number of selected features. However, for the vast majority of the features, using all the features cannot obtain the optimal recognition rate. This means that not all the features are beneficial for the identification. Therefore, the number of selected features corresponding to the best classification accuracy is regarded as the optimal number of the subset after feature selection.

We can obtain the optimal numbers of different kinds of features with COS and COR distance metrics when *k* = 1 for both Datasets, which are given in Table 7 and Table 8.

### 4.4. Comparison of Classification Accuracies with and without Feature Selection

Table 9 lists the classification results of Dataset I with/without feature selection, and Table 10 lists the classification results of Dataset II with/without feature selection. It can be concluded from the Table 9 and Table 10 that the classification accuracy with feature selection based on the proposed feature selection approach is improved obviously than without feature selection. For Dataset I, it can be seen that the Db1 and Db2 achieve best classification accuracy when the distance metric is COR with feature selection, which achieve 96.25%. For Dataset II, the classification accuracies of DR and EMAi3 can achieve the classification rate above 70% when the distance metric is COR. However, for COS, only the classification accuracies of DR can achieve the classification rate above 70%. Hence, COR is used as the optimal distance metric in the following feature fusion framework.

### 4.5. Results of Feature Fusion

**Figure 6:**
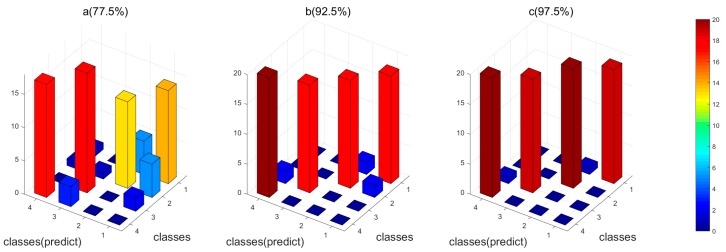
The 3D plot of classification results of the four feature fusion methods for Dataset I. (**a**) The conventional feature fusion method without feature selection; (**b**) the proposed feature fusion method without feature selection; (**c**) the proposed feature fusion method with feature selection; (Note: 1, No infection; 2, *S. aureus*; 3, *E. coli*; 4, *P. aeruginosa*.).

**Figure 7:**
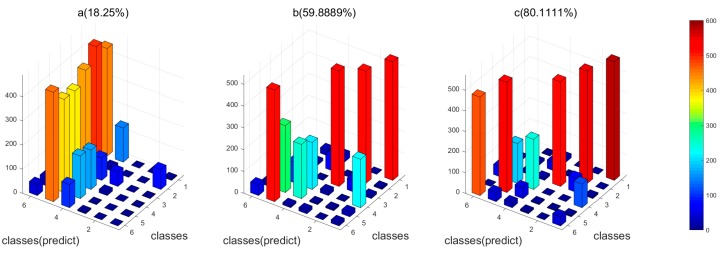
The 3D plot of classification results of the four feature fusion methods for Dataset II. (**a**) The conventional feature fusion method without feature selection; (**b**) the proposed feature fusion method without feature selection; (**c**) the proposed feature fusion method with feature selection; (Note: 1, ethanol; 2, ethylene; 3, ammonia; 4, acetaldehyde; 5, acetone; 6, toluene).

In order to compare the performance of different identification methods, we applied two control methods: (1) the conventional feature fusion strategy combining the original features simply and directly; (2) the proposed feature fusion method but without feature selection. Figure 6 and Figure 7 showed the results of the three methods for both the datasets, respectively. For both of the datasets, the proposed feature selection and fusion method can obtain the best classification accuracy. Particularly, for Dataset I, it is observed that the conventional feature fusion method, which integrates all the features without feature selection, only has the accuracy of 77.5%, which is much lower than the other two methods. The conventional fusion method does not consider the importance of each kind of feature, which have distinct contributions to the identification. The proposed feature fusion method enhances the effects of “good” features and suppresses the effects of “bad” features, which can improve the performance. The proposed feature selection and fusion method can obtain the highest accuracy of 97.5% among the three methods. It demonstrates that the redundant features will deteriorate the performance of the gas sensor array, and the proposed feature selection methods can eliminate the redundant and irrelevant features and finally enhance the performance of the gas sensor array. For Dataset II, we can see clearly in Figure 7 that the recognition rate of the proposed feature fusion method with selected features obviously improved the performance, which has achieved an accuracy of 80.11%. The classification for each kind of gas has been significantly improved, compared with the conventional method. This means that the proposed feature selection and fusion method can effectively compensate the drift effect and enhance the discrimination ability of the gas sensor array.

## 5. Conclusions

In this paper, efforts are made to improve the discrimination ability of a gas sensor array using a new feature selection and feature fusion framework. The feature selection integrates the filter and the wrapper approaches, and the feature selection emphasizes the classification dominance fusion strategy based on the classification rates of each base feature. Compared with original features, the selected features can better represent the E-nose signal characteristics. Based on the proposed framework, the E-nose performs better markedly than not only using all the basis features without selection, but also the conventional feature fusion method. The experimental results show that the classification rates of gases have been excellent, and improved after feature selection compared with the results without feature selection. The feature selection method can select more relevant but less redundant feature elements, which are beneficial to the gas identification. Furthermore, compared with the conventional direct fusion method, the fusion method proposed in this paper has better performance on gas classification, as the conventional fusion method directly integrates “good” features as well as “bad” features to one blended feature matrix, without considering the different discrimination ability of each kind of features. The blended feature matrix may contain redundancies between feature elements, which are not conducive for distinguishing different kinds of gases. Moreover, the discrimination ability of the base feature is the most intuitive indicator of the contribution of each feature for the final discrimination in the decision level. It indicates that the superiority of the proposed feature selection and fusion framework in enhancing E-nose performance. It also indicates that proposed framework can be successfully used in overcoming the long-term drift effect of a gas sensor array.

## Figures and Tables

**Table 1:** Number of samples in Dataset I.

Group	Training Set	Test Set
No infection	20	20
*Pseudomonas aeruginosa*	20	20
*Escherichia coli*	20	20
*Staphylococcus aureus*	20	20
Total	80	80

**Table 2:** Data structure of seven features.

Features	MV	FFT	Db1	Db2	Db3	Db4	Db5
Feature structure	15 × 80	30 × 80	30 × 80	60 × 80	90 × 80	120 × 80	150 × 80

Note: MV, maximum value; FFT, the DC component and first order harmonic component of the coefficients of fast Fourier transformation; Db1, Db2, Db3, Db4, Db5, the approximation coefficients of discrete wavelet transformation based on wavelets Db1, Db2, Db3, Db4, and Db5, respectively.

**Table 3:** Concentration ranges of analytes in Dataset II.

Analytes	Ammonia	Acetaldehyde	Acetone	Ethylene	Ethanol	Toluene
Concentration Range (ppm)	50–1000	5–300	10–300	10–300	10–600	10–100

**Table 4:** Experimental long-term sensor drift big data.

Batch ID	Month	Number of the Data					
		Ethanol	Ethylene	Ammonia	Acetaldehyde	Acetone	Toluene
Batch 1	1, 2	83	30	70	98	90	74
Batch 2	3~10	100	109	532	334	164	5
Batch 3	11, 12, 13	216	240	275	490	365	0
Batch 4	14, 15	12	30	12	43	64	0
Batch 5	16	20	46	63	40	28	0
Batch 6	17~20	110	29	606	574	514	467
Batch 7	21	360	744	630	662	649	568
Batch 8	22, 23	40	33	143	30	30	18
Batch 9	24, 30	100	75	78	55	61	101
Batch 10	36	600	600	600	600	600	600

**Table 5:** Classification results of seven features based on different values of *k* and distance metrics for Dataset I (%).

Distance	*k*	MV	FFT	Db1	Db2	Db3	Db4	Db5
EU	1	68.75	73.75	90.00	**91.25**	87.50	88.75	83.75
	3	63.75	72.50	75.00	78.75	78.75	81.25	80.00
	5	46.25	45.00	66.25	70.00	70.00	71.25	73.75
	7	51.25	53.75	60.00	68.75	70.00	73.75	75.00
	9	43.75	60.00	57.50	66.25	66.25	66.25	70.00
CB	1	66.25	70.00	**91.25**	**91.25**	86.25	86.25	82.50
	3	60.00	62.50	71.25	75.00	76.25	77.50	75.00
	5	41.25	47.50	61.25	71.25	70.00	72.50	72.50
	7	52.50	53.75	57.50	71.25	71.25	72.50	73.75
	9	48.75	48.75	55.00	65.00	63.75	65.00	67.50
COS	1	77.50	77.50	90.00	**92.50**	91.25	91.25	86.25
	3	72.50	78.75	80.00	82.50	82.50	82.50	82.50
	5	57.50	60.00	68.75	68.75	72.50	73.75	80.00
	7	58.75	53.75	63.75	65.00	67.50	71.25	75.00
	9	46.25	43.75	55.00	62.50	61.25	61.25	66.25
COR	1	78.75	77.50	**93.75**	92.50	91.25	92.50	87.50
	3	71.25	76.25	81.25	85.00	85.00	85.00	85.00
	5	56.25	57.50	66.25	76.25	75.00	76.25	82.50
	7	52.50	61.25	63.75	66.25	67.50	71.25	76.25
	9	51.25	58.75	50.00	58.75	63.75	65.00	67.50

Note: EU, Euclidean distance; CB, cityblock distance; COS, cosine distance; COR, correlation distance. The bold numbers are the highest accuracies for each distance metric.

**Table 6:** Classification results of eight features based on different values of *k* and distance metrics for Dataset II (%).

Distance	*k*	DR	NDR	EMAi1	EMAi2	EMAi3	EMAd1	EMAd2	EMAd3
EU	1	53.53	**60.00**	36.31	53.61	59.06	36.31	43.56	48.78
	3	54.25	58.89	37.28	54.03	58.42	36.61	43.47	49.39
	5	54.06	59.42	38.47	54.28	58.00	37.11	43.47	49.00
	7	53.47	59.53	38.61	54.08	57.97	37.39	43.25	48.42
	9	53.50	59.50	38.11	53.64	57.61	37.03	43.00	48.31
CB	1	57.33	61.97	38.22	54.03	60.25	36.64	44.36	50.50
	3	60.58	**62.19**	37.50	55.78	58.89	36.75	44.42	51.36
	5	60.00	61.28	38.69	55.58	59.36	36.81	44.31	51.28
	7	60.03	61.78	38.11	54.53	59.61	37.03	44.36	51.03
	9	59.97	62.17	37.86	53.69	60.28	36.89	44.31	51.50
COS	1	49.42	**60.42**	37.39	52.22	57.25	34.50	43.39	48.58
	3	51.33	59.81	37.97	50.47	55.28	35.00	43.56	48.69
	5	51.31	59.22	38.28	50.42	55.58	35.64	43.19	48.50
	7	51.53	59.31	38.08	50.28	55.42	36.17	42.94	48.42
	9	52.00	59.00	37.78	50.06	55.42	36.19	42.58	48.19
COR	1	49.56	59.72	37.89	51.03	56.44	35.72	40.86	46.69
	3	49.94	**59.97**	37.75	49.50	54.58	35.61	41.06	47.31
	5	50.25	59.53	37.86	49.94	55.00	35.08	40.56	47.78
	7	50.36	59.14	37.36	49.11	54.69	35.67	40.47	47.50
	9	50.22	59.28	36.97	48.81	55.69	36.28	40.14	47.14

Note: the bold numbers are the highest accuracies for each distance metric.

**Table 7:** Optimal numbers of different features after selection for Dataset I.

Features		MV	FFT	Db1	Db2	Db3	Db4	Db5
Distance metrics	COS	15	18	21	10	10	36	74
	COR	14	26	25	23	18	49	109

**Table 8:** Optimal numbers of different features after selection for Dataset II.

Features		DR	NDR	EMAi1	EMAi2	EMAi3	EMAd1	EMAd2	EMAd3
Distance metrics	COS	13	16	7	13	8	7	11	12
	COR	13	9	7	10	8	16	11	12

**Table 9:** Classification accuracy of Dataset I with/without feature selection.

Features			MV	FFT	Db1	Db2	Db3	Db4	Db5
Without selection	COS	Dimension	**15**	**30**	**30**	**60**	**90**	**120**	**150**
		1	80.00	85.00	90.00	90.00	95.00	95.00	95.00
		2	80.00	80.00	90.00	95.00	85.00	85.00	80.00
		3	65.00	70.00	95.00	90.00	90.00	90.00	90.00
		4	85.00	75.00	85.00	95.00	95.00	95.00	80.00
		Average	77.50	77.50	90.00	92.50	91.25	91.25	86.25
	COR	1	75.00	85.00	95.00	90.00	90.00	95.00	95.00
		2	85.00	85.00	100.00	90.00	90.00	90.00	85.00
		3	70.00	65.00	90.00	90.00	90.00	90.00	90.00
		4	85.00	75.00	90.00	100.00	95.00	95.00	80.00
		Average	78.75	77.50	93.75	92.50	91.25	92.50	87.50
With selection	COS	Dimension	**15**	**18**	**21**	**10**	**10**	**36**	**74**
		1	80.00	85.00	95.00	95.00	95.00	95.00	95.00
		2	80.00	80.00	90.00	90.00	95.00	90.00	85.00
		3	65.00	75.00	95.00	95.00	90.00	95.00	85.00
		4	85.00	80.00	95.00	95.00	90.00	90.00	90.00
		Average	77.50	80.00	93.75	93.75	92.50	92.50	88.75
	COR	Dimension	**14**	**26**	**25**	**23**	**18**	**49**	**109**
		1	85.00	85.00	95.00	95.00	90.00	90.00	95.00
		2	90.00	85.00	100.00	95.00	95.00	100.00	85.00
		3	70.00	70.00	95.00	95.00	95.00	95.00	90.00
		4	80.00	80.00	95.00	100.00	95.00	95.00	85.00
		Average	81.25	80.00	**96.25**	**96.25**	93.75	95.00	88.75

Note: 1, No-infection; 2, *S. aureus*; 3, *E. coli*; 4, *P. aeruginosa*.

**Table 10:** Classification accuracy of Dataset II with/without feature selection.

Features			DR	NDR	EMAi1	EMAi2	EMAi3	EMAd1	EMAd2	EMAd3
without selection	COS	Dimension	**16**	**16**	**16**	**16**	**16**	**16**	**16**	**16**
		1	61.00	26.00	73.17	98.67	65.00	61.17	69.67	85.00
		2	85.17	98.67	67.17	83.33	84.50	54.00	62.83	67.67
		3	90.33	91.83	10.00	37.17	89.33	27.33	58.50	79.50
		4	6.67	17.33	40.67	59.50	67.67	1.33	5.83	15.00
		5	46.17	58.17	30.83	23.83	27.50	23.33	55.50	40.67
		6	7.17	70.50	2.50	10.83	9.50	39.83	8.00	3.67
		Average	49.42	60.42	37.39	52.22	57.25	34.50	43.39	48.58
	COR	1	53.00	25.50	73.33	98.83	65.50	60.83	70.67	83.00
		2	85.17	98.83	67.67	83.83	83.83	54.33	63.17	68.67
		3	90.17	90.17	6.33	29.50	86.33	27.33	45.67	78.67
		4	5.17	14.83	43.67	58.17	67.67	0.83	4.50	11.50
		5	51.83	59.83	32.17	22.67	24.00	20.67	51.50	34.67
		6	12.00	69.17	4.17	13.17	11.33	50.33	9.67	3.67
		Average	49.56	59.72	37.89	51.03	56.44	35.72	40.86	46.69
with selection	COS	Dimension	**13**	**16**	**7**	**13**	**8**	**7**	**11**	**12**
		1	47.17	26.00	75.00	87.50	82.00	52.50	77.83	82.50
		2	92.00	98.67	67.17	84.33	90.17	53.00	66.00	79.33
		3	85.83	91.83	31.83	32.83	75.33	31.67	58.67	76.50
		4	31.67	17.33	16.50	71.33	43.00	1.50	30.50	20.00
		5	93.67	58.17	88.17	49.33	62.83	41.00	70.00	47.00
		6	76.33	70.50	19.67	29.50	59.67	43.67	47.17	62.00
		Average	**71.11**	60.42	49.72	59.14	68.83	37.22	58.36	61.22
	COR	Dimension	**13**	**9**	**7**	**10**	**8**	**16**	**11**	**12**
		1	41.00	51.00	73.33	92.17	85.83	60.83	74.00	79.50
		2	92.67	99.17	72.33	87.50	91.17	54.33	64.00	79.33
		3	86.67	98.67	24.17	60.50	59.33	27.33	44.83	76.67
		4	31.33	0.00	9.50	5.33	43.67	0.83	16.00	9.33
		5	93.17	30.33	81.83	43.33	65.33	20.67	69.00	42.17
		6	81.33	95.67	37.17	52.83	91.00	50.33	47.17	60.33
		Average	71.03	62.47	49.72	56.94	**72.72**	35.72	52.50	57.89

Note: 1, ethanol; 2, ethylene; 3, ammonia; 4, acetaldehyde; 5, acetone; 6, toluene.
